# Supplementary material for: Dynamics of Replication-Associated Protein Levels through the Cell Cycle
Source: Int J Mol Sci. 2024 Jul 28;25(15):8230. doi: 10.3390/ijms25158230 (PMC11311332; doi:10.3390/ijms25158230)
Supplement: Supplementary file 1 [file ijms-25-08230-s001.zip › ijms-3099532-SM-main.pdf]

Figure S1

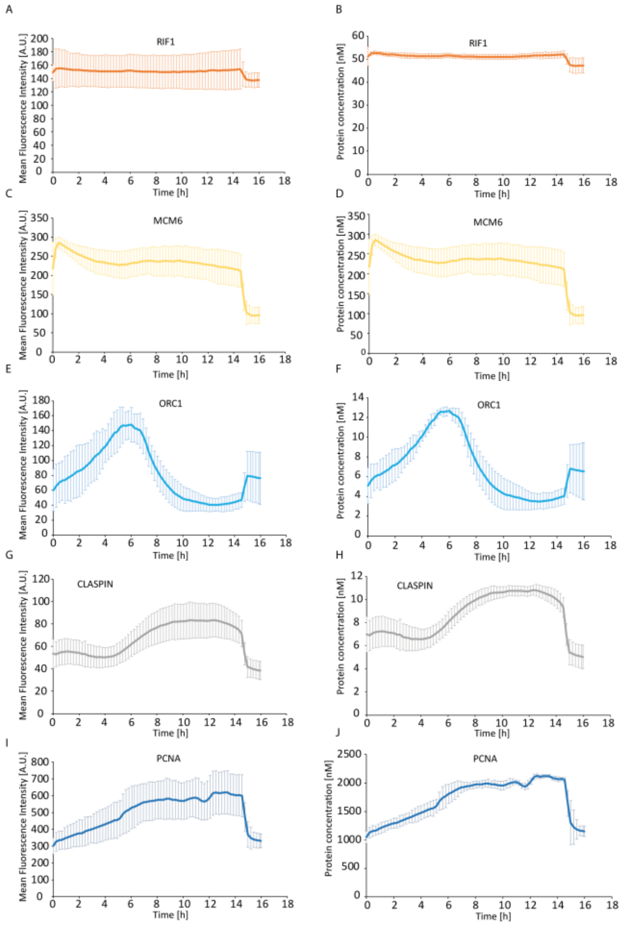

Figure S1. Changes in mean fluorescence intensity and protein concentration of RIF1 (A, B), MCM6 (C, D), ORC1 (E, F), Claspin (G, H), and PCNA (I, J) through the cell cycle. Data are presented as mean ± SD.

Figure S2

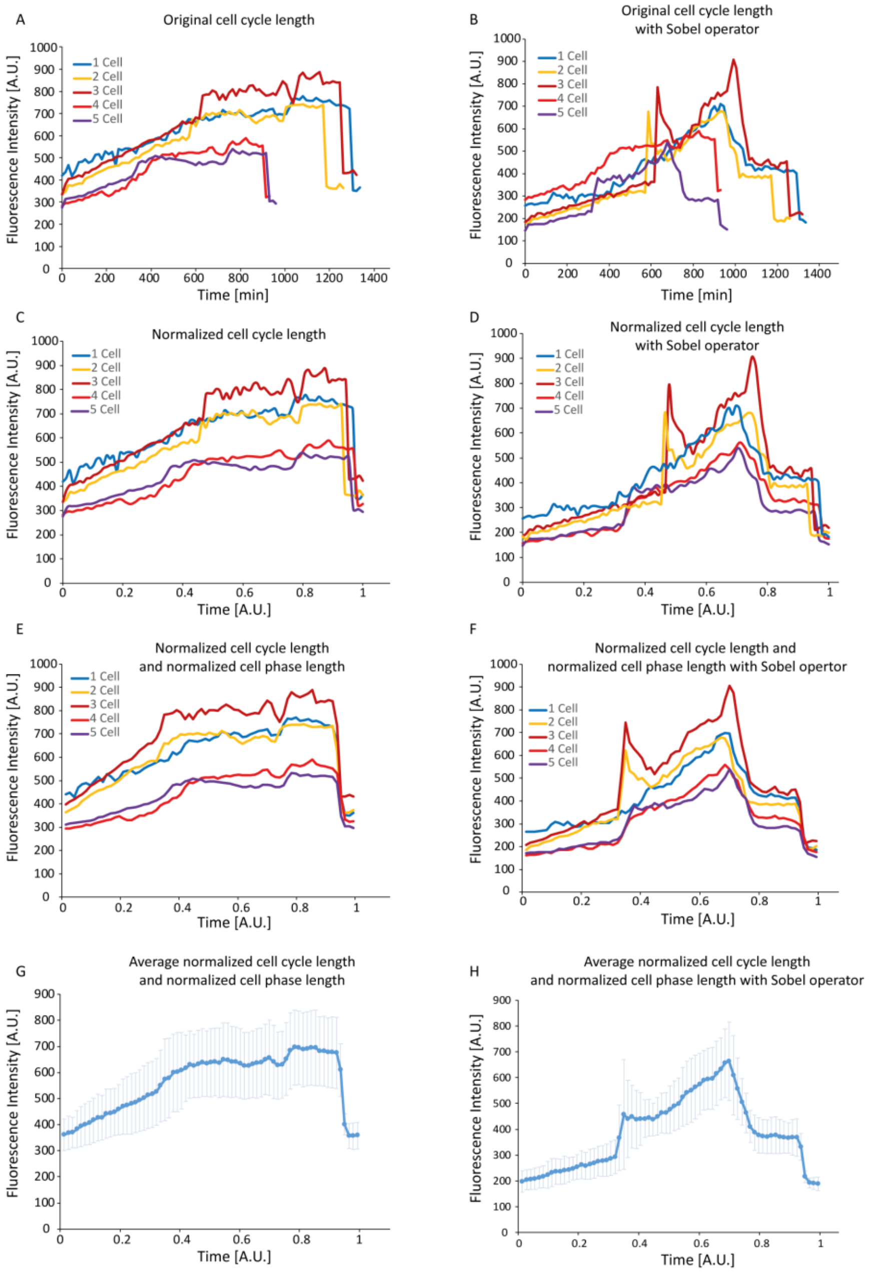

Figure S2. Cell cycle and cycle phase length normalization. (A) Fluorescence intensity of five PCNA-mCherry HeLa Kyoto cells with different cell cycle lengths. (B) Sobel-adjusted intensity for the cells in (A). (C) Fluorescence intensity of the cells from (A) with a normalized cell cycle length (0–1). (D) Sobel-adjusted intensity over the normalized cell cycle length. (E) Fluorescence intensity of the cells from (A) with normalized cell cycle and cycle phase lengths. (F) Sobel-adjusted fluorescence intensity of the cells from (A) with normalized cell cycle and cycle phase lengths. (G) Average fluorescence intensity with normalized cell cycle and cycle phase length (n =10 cells). (H) Average Sobel-adjusted fluorescence intensity with normalized cell cycle and cycle phase length (n = 10 cells). Data are presented as mean ± SD.
